# CCR5: From Natural Resistance to a New Anti-HIV Strategy

**DOI:** 10.3390/v2020574

**Published:** 2010-02-05

**Authors:** Lucia Lopalco

**Affiliations:** Division of Immunology, Transplantation and Infectious Diseases, San Raffaele Scientific Institute, via Stamira D’Ancona, 20, 20127 Milan, Italy; E-Mail: lopalco.lucia@hsr.it; Tel.: +39-02-2643-7936; Fax: +39-02-2643-5381

**Keywords:** CCR5, HIV, vaccine

## Abstract

The C-C chemokine receptor type 5 (CCR5) is a key player in HIV infection due to its major involvement in the infection process. Investigations into the role of the CCR5 coreceptor first focused on its binding to the virus and the molecular mechanisms leading to the entry and spread of HIV. The identification of naturally occurring CCR5 mutations has allowed scientists to address the CCR5 molecule as a promising target to prevent or limit HIV infection *in vivo*. Naturally occurring CCR5-specific antibodies have been found in exposed but uninfected people, and in a subset of HIV seropositive people who show long-term control of the infection. This suggests that natural autoimmunity to the CCR5 coreceptor exists and may play a role in HIV control. Such natural immunity has prompted strategies aimed at achieving anti-HIV humoral responses through CCR5 targeting, which will be described here.

## 1. Introduction

More than 40 million people, mostly women and children, are presently infected by the human immunodeficiency virus (HIV); almost all horizontal and vertical transmissions of HIV infection are due to HIV strains that use the CCR5 coreceptor expressed on mucosal surface [1,2].

CCR5 is undoubtedly the main HIV coreceptor, involved in virus entry and cell-to-cell spread: Such R5-tropic viruses are nearly always involved in the initial infection, while HIV strains using the CXCR4 coreceptor are observed only seldomly in the early infection [3]. Due to the natural history of HIV infection, CCR5 is a key target for the development of drugs and immunogens that are able to elicit systemic and especially mucosal responses to protect exposed people from infection. Easy-to-use, cheap, and long-lasting mucosal protection could significantly limit HIV spread, especially in sub-Saharan Africa, Eastern Asia, and other areas where sexually transmitted diseases are heavy health and social burdens [4].

The discovery of CCR5 genetic polymorphisms associated with HIV-resistance or disease control encouraged the research and development of inhibitor-drugs and antibodies that are able to counteract HIV at its major portal of entry; some of these products are presently undergoing evaluation in clinical trials or have even been licensed for therapy [5,6]. Stemming from such CCR5 investigations, natural anti-CCR5 immunity was observed in special populations dealing with HIV, *i.e.*, HIV-exposed seronegative people and long-term non-progressing seropositive individuals [7]. Strikingly, such antibodies ─ found in serum, but most importantly also in mucosal secretions ─ were associated with a protective role or with control of the disease [8,9].

These observations confirm that CCR5 is a promising target in the prevention or therapy of HIV, and suggest that even innovative approaches, such as anti-CCR5 vaccination, can provide useful scientific insight; and but most importantly, valuable weapons to fight HIV and other immune-based diseases. This review will discuss the role of CCR5 in HIV infection and the current approaches to target CCR5, with particular attention to the cases of natural immunity to the coreceptor and immunization experiments aimed at reproducing it.

## 2. CCR5 functions

**Figure 1.**
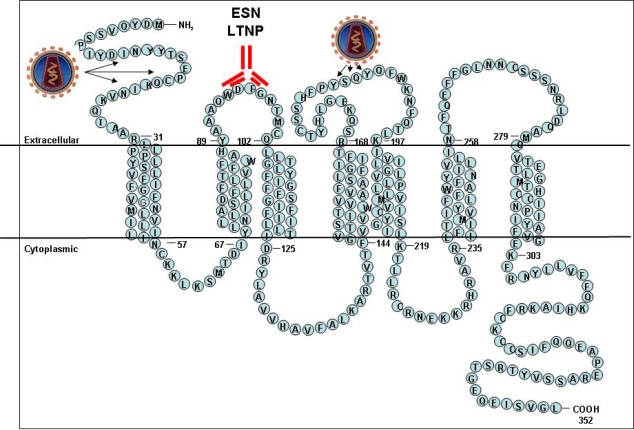
Structure of the CCR5 coreceptor; the HIV binding domains (N-terminal and ECL2 domain) and the ECL1 domain are indicated.

CCR5 belongs to a large family of chemokine receptors that are expressed on surface of lymphocytes and other cell types, where they are involved in signaling and coordination of immune responses [10]. Similarly to CXCR4, CCR5 is also an HIV coreceptor [11–14]. CCR5 and other chemokine receptors belong to an even larger family of seven transmembrane proteins coupled to G proteins, a very important family that includes many signaling receptors, such as rhodopsin and beta-adrenergic receptors [10]. Seven transmembrane receptors are large molecules (Figure 1), however their three-dimensional structures are still poorly elucidated from physico-chemical spectroscopic methods, such as X-ray crystallography. Only the structure of rhodopsin, the two beta adrenergic receptors, and the adenosine receptor have been recently characterized [1]. The approximate structures of other receptors, such as CCR5, have been modeled based on similarities revealed by the structures of these related proteins [1].

CCR5 is expressed on immature (Th0) and memory and primed Th1 cells, monocytes, macrophages, and immature DC; on neurons, astrocytes, and microglia; on epithelium, endothelium, vascular smooth muscle, and fibroblasts [2]. Its preferential ligands are the pro-inflammatory cytokines CCL3 (MIP-1 alpha), CCL4 (MIP-1beta) and CCL5 (RANTES), involved in the initiation of effector responses [3]. Other cytokines, such as CCL7 (MCP-3), CCL8 (MCP-2) and CCL13 (MCP-4), are a competitive antagonist and two weak agonists, respectively. Since chemokine binding may interfere with HIV docking, natural CCR5 ligands were evaluated as HIV competitors, with varying results: CCL3, CCL4, CCL5 and CCL8 displayed inhibiting properties to HIV, CCL7 was shown not to interfere, while CCL2 (MCP-1) even enhanced HIV infection *in vitro* [4]. CCL3L1 and CCL4L1 are variant chemokines encoded by genes with varying copy numbers. These chemokines inhibit the binding of CCR5 to HIV through receptor down-regulation, in an inverse relationship; their gene copy numbers and hence the expression levels influence HIV progression [5].

CCR5 is not only a chemokine receptor that is able to induce cell chemotaxis towards chemokine gradients, but it also takes part in immune synapses where it behaves as a costimulatory molecule [6]. More specifically, CCR5 is involved in the orchestration of cellular immunity, which is a CCL5/RANTES-mediated cascade that is independent of the chemotactic response [7]. CCL5 was shown to induce the expression of activation markers at the surface of primary T cells *in vitro; in vivo*, it increased the proliferative response to antigens in CD4+ T cells and subsequent cytokine secretion [6]. CCR5 was also shown to sustain recruitment of naive CD8+ T cells to antigen-presenting dendritic cells [8]. CCR5 chemokine ligands can enhance effector responses by potentiating APC and T cell activities in response to antigen-induced stimulation [9].

The mechanism of CCR5 signaling first requires the ligand binding to the extracellular domains of the receptor, followed by receptor dimerization and phosphorylation [10]. Intracellular signal transduction, mediated by GDP release, requires binding and hydrolysis of a new GTP molecule [1]. The activated G protein then dissociates from the cytoplasmic domain of CCR5 and activates a second-messenger cascade, sustained by phospholipase C kinase, inositol-triphosphate (IP3-kinase) and mitogen-activated (MAP) kinases or other tyrosine kinases [11]. The mechanism of CCR5 signaling and regulation is complex, and most aspects are still not well understood. Experiments aimed at correlating CCR5 structure and function by using monoclonal antibody panels suggests that CCR5 internalization may occur via phosphorylation and binding to arrestin before internalization in clathrin-coated vesicles, as observed upon chemokine stimulation [12]. However, CCR5 internalization may also involve a different pathway, dependent on cholesterol-rich membrane caveolae [13]. CCR5 was identified in membrane raft microdomains and its subcellular localization was supposed to contribute to chemotaxis as well as to HIV entry [14]. However, constitutive CCR5 turnover also occurs in the absence of ligand, with a half-life of six to nine hours [15]. Thr in-and-out flux of CCR5 molecules is highly regulated as surface receptor density is inversely correlated with CCL5/RANTES expression levels [16].

CCR5 could be anchored to plasma membrane lipid rafts through the palmitoylated cysteine residues located in its C-terminal domain [17]. CCR5 molecules placed in the rafts closely cluster with CD4 on the cell surface, therefore providing a convenient “docking site” for HIV [18]. CD4 and CCR5 were not only found physically associated as HIV coreceptors [19,20], but CD4 was also shown to associate with CCR5 molecules from the endoplasmic reticulum, thus promoting their exposure at the cell surface [21].

Wild-type CCR5 is able to polymerize, not only with itself [22], but also with its truncated delta-32 form [23], with other chemokine receptors, such as CCR2 [24], and even with other GPCR, such as the opioid receptor [25]. The biological significance that homo- and hetero-dimerization has on CCR5 conformation, binding, and signaling are presently unknown.

## 3. CCR5 deletion and its consequences

CCR5 expression levels may vary in individuals without affecting immune function [26]. Depending on the number of exposed receptors, low and high “CCR5 expression” individuals have been described [27]. This variation in CCR5 expression levels between individuals reflect genetic factors as well as environmental stimuli, as reported in a comparative study that observed higher levels of CCR5 expression and immune activation in European and African subjects residing in Africa ─ possibly due to parasitic infections ─ than in a cohort of the same ethnic groups residing in Europe [28]. Genetic patterns that prevent CCR5 expression have been described in HIV-exposed uninfected people who display natural resistance to HIV infection [29,30]; also, the enhanced expression of chemokines has been reported to play a role in natural resistance to HIV [31]. Reduced or abolished expression of the CCR5 receptor has been found in Caucasians and in other ethnic groups worldwide; the delta-32 mutation - the first to be described - causes a deletion in the receptor sequence that prevents exposure of the truncated receptor on the cell surface [4]. Consequently, homozygous delta-32 individuals are substantially ─ but not completely ─ resistant to HIV infection, but do not show any pathologic phenotype [32,33,34]. In some cases, infection of delta-32 homozygous individuals was associated with dual tropic R5-X4 or to X4-tropic viral strains [35,36,37]. As confirmation of delta-32 resistance to HIV, a recent clinical observation showed long-term control of infection without antiretroviral therapy in an HIV-positive patient who had the CCR5+ genotype and underwent CCR5-/- stem cells transplantation to treat acute myeloid lymphoma [38]. CCR5+ macrophages were identified in a patient biopsy from intestinal mucosa, taken several weeks after transplantation, showing that the circulating, but not the resident CCR5+ cells, had been replenished by the transplant. However, viral RNA was undetectable despite the persistence of HIV-permissive cells, and the patient did not experience a rebound in viral load in the absence of antiretroviral therapy. Another important finding of the study was the absence of a virus shift in favor of X4 strains, whose presence was unnoticed by current diagnostics practices, but was, however, detected by ultra-deep sequencing [38]. Heterozygous CCR5-delta-32 alleles have been found to be more prevalent in long-term non-progressing population than in progressing cohorts, therefore confirming that CCR5 load and functions may play a more complex role than that of coreceptor in the pathogenesis of HIV infection [39,40]. Some hypotheses have been drawn to explain the evolution and the selective advantage conferred by CCR5 delta-32 allele in humans, such as an increased resistance to plague or smallpox, but none is presently conclusive [41].

Other CCR5 mutations have been subsequently described; interestingly, high levels of circulating beta-chemokines (e.g., CCL5/RANTES) also affects HIV binding to CCR5 molecules [26]. Both events converge on the CCR5 receptor, but different mechanisms may be involved in each of these. In fact, homozygous CCR5 mutation may prevent wild-type CCR5 from being exposed on the cell surface; circulating chemokines can compete with HIV for binding, mask the viral binding site, or subtract the whole receptor from the cell surface by inducing its internalization [4]. A large cohort study, involving over 2000 HIV-positive and healthy people, compared the two major parameters of clinical status in HIV infection, *i.e.*, viral load and CD4+ T cell counts, and two parameters representing immune activation and inflammation, *i.e.*, CCR5 expression and gene copy number of the CCL3-L1 molecule, a natural cytokine acting as the most powerful CCR5 inhibitor *in vivo*. The study population was stratified according to CCR5 expression level (high *vs*. low) and CCL3L1 genotype (high copy number *vs*. low copy number), and genetic profiling defined individuals with high-, moderate-, or low-risk of HIV-progression. However, CCR5 expression and these clinical parameters were not strongly associated, suggesting that CD4+ T cell depletion is not only due to the rates of HIV infection and replication (and therefore to CCR5 expression), but also to other immune mechanisms, such as cell-mediated immunity (CMI). The exact mechanism(s) leading to CMI impairment is presently undetermined, but it is expected to exert more subtle effects than the mere down-regulation of the CCR5 receptor or the competition with HIV binding [42]. CCR5 knockout mice (ccr5-/-) were found to develop normally, and also showed a more robust T-cell response to a number of antigens than wild-type (WT) mice [43]. When ccr5-/- mice were experimentally infected with West Nile Virus (WNV), all of them succumbed to the infection, while the majority of WT mice survived. CCR5-deficient mice failed to control virus replication in the CNS, due to reduced recruitment of infiltrating CD4+ and CD8+ T cells, NK cells, and macrophages, suggesting a disequilibrium between pathogen immunity and deleterious effects of inflammation [44]. In the experimental infection with HSV-2, ccr5 -/- mice also showed higher brain titers than WT control animals, but were able to clear the infection [45]. Similar results were observed in cohort studies on patients infected by WNV, which showed an increased risk of symptomatic infection in homozygous carriers of the delta-32 mutation [46]. Other flavivirus infections, such as the tick-borne encephalitis (TBE), an endemic infection in Europe and Asia, were found to be associated with delta-32 alleles [41].

CCR5 also takes part in the response to bacteria and bacterial products, such as lipopolysaccharide (LPS) and heat-shock proteins. Macrophages from CCR5-deficient mice challenged with LPS show an impaired function; this finding was associated with a reduced efficiency in clearance of *Listeria* infection and with a protective effect against LPS-induced endotoxemia [43]. The possible association of CCR5 deficiency with other diseases, such as hepatitis C, and with autoimmune disorders, such as multiple sclerosis, has not been proven [47]. However, CCR5 deficiency was shown to play a protective role in rheumatoid arthritis [48], supporting the use of CCR5 antagonists in clinical treatment of autoimmune, inflammation-based disorders. In this case, CCR5 blockage may inhibit T cell migration, a key pathway in the inflammatory process causing pain, tissue damage, and disability [49]. Acute rejection is characterized by cell recruitment into clinical allografts via CCR5-mediated cytokine signaling; for instance, immunosuppressed patients receiving renal transplants who are homozygous carriers of the CCR5 delta-32 allele rarely exhibit late graft loss. The use of cyclosporine A in association with a CCR5 inhibitor reduces leukocyte recruitment to grafts and prolongs their survival in a cynomolgus model of monkey cardiac allograft model [50].

## 4. CCR5 role in HIV infection

HIV entry engages the viral *env* glycoprotein complex, the CD4 antigen, and a chemokine receptor, nearly always CCR5 ─ sometimes CXCR4, especially in later stages of disease ─ both located on the surface of the host cell. The virus envelope consists of two proteins, gp120 and gp41, which mediate virus attachment on the host cell, binding, and fusion with the target cell membrane. The external gp120 and the transmembrane gp41 subunits are generated by proteolytic cleavage of a larger precursor, gp160, and are not covalently associated; three *env* complexes form trimeric spikes on the virus particle. Although the three-dimensional structure of the *env*-receptor complex has not been fully elucidated by spectrometric analysis, biochemical, genetic, and immunological investigations have provided information about the order of event and the protein domains taking part in it [51].

Binding of gp120 to CD4 generates a conformational change in the *env* complex and exposes ─ or induces ─ the CCR5 binding site, whose major domains are the bridging sheet and the variable V3 loop. *Env* domains interacting with the N-terminus and the second extracellular loop of CCR5 cause a conformational change in the coreceptor, which activates the coreceptor signaling. Conversely, CCR5 binding triggers further conformational changes, leading to the extension of the gp41 fusogenic domain and to refolding of the gp41 trimer in a six-helix bundle, bringing lipid bilayers into close contact and eventually leading to fusion [4]. Comparative studies employing monoclonal antibody panels, chimeric molecules, viral pseudotypes or site-directed mutagenesis, have helped to understand the key determinants of binding. HIV binding has been shown to involve the N-terminus and the second extracellular loop of the CCR5 molecule, while natural CCR5 ligands, such as CCL4/MIP-1beta or CCL5/RANTES, bind to overlapping regions on the receptor, different for each ligand, and compete for binding with the virus. Some monoclonal antibodies were also found to promote receptor signaling and internalization, mediated by a conformational change requiring CCR5 oligomerization [52]. However, HIV binding may occur with wild-type and even with C-truncated CCR5 receptors, which are unable to be internalized or to transduce signaling to G proteins, therefore showing that this event is not required for efficient cell infection [53–55]. Direct crystallographic approaches, as well as indirect biochemical or immunological studies, have led the way in the design and synthesis of drugs targeting CCR5, such as Maraviroc, which was approved for clinical use in 2007 [56].

### 4.1. CCR5 *vs.* CXCR4

Dendritic cells (DC) are natural sentinel cells that sample incoming pathogens or their antigens at the mucosal epithelia, transport them to regional lymph nodes, and there present them to T and B cells in order to initiate adaptive immune responses [57]. DC express CCR5, but not the CXCR4 receptor, and therefore are exposed to infection by R5 virus strains. Such strains preferentially penetrate mucosal barriers, leading to lymph nodes drainage, by using DC as Trojan horses [58]. Indeed, when infected DC prime and activate CD4+ T cells within lymph nodes, the virus is placed in a perfect environment that favors its rapid and efficient amplification, and R5 viruses dominate the scene because of the expression of CCR5 on activated CD4+ T cells [59].

Extremely high levels of replication of SIV can occur in naturally infected monkeys without the onset of immunodeficiency [60]. In HIV-infected humans, immune decline is associated with viral impairment of the regenerative capacity of the defense system [61]. X4 viral strains can cause local damage to the thymus by targeting developing T cells there. R5 viruses are less cytopathic than X4 strains in cultured thymocytes; they also replicate in the thymus *in vivo*, but without damaging the developing T cells; In fact, CCR5 expression is lower than CXCR4 expression during T cell development [62]. For instance, children carrying X4 viruses show a greater impairment of thymic function and CD4+ T cells than those infected with R5 viruses [63]. Immune activation due to HIV infection causes CCR5 up-regulation in CD4+ cells [64]. Overall, the relative expression levels of CCR5 and CXCR4 in PBMC do not influence the rate of evolution of X4 variants; the availability of CXCR4+ cells does not increase the evolution rate of X4 viruses; moreover, CXCR4 usage is not an escape mechanism adopted to overcome propagation limits due to a lower count of CCR5+ target cells [65]. The lack of a fast progression to AIDS due to expansion of X4 viruses in most individuals could be due to virological and to immunological reasons. On one hand, R5 viruses are favored by the prompt and abundant availability of CCR5+ cells in mucosae, in professional APC, and in lymph nodes. On the other hand, the emergence of X4 strains in late stages of infection might reflect the progressive accumulation of immune damage [66].

### 4.2. CCR5 in mucosal HIV transmission

Clinical observations confirm that mucosal transmission of HIV is nearly exclusively due to CCR5-dependent HIV strains [67]. Dual-tropic, R5X4 viruses, or the rarer CXCR4-dependent viruses, are observed in late phases of the infection, and are usually associated with a faster progression to AIDS and to a marked decline of immune response [66].

**Figure 2.**
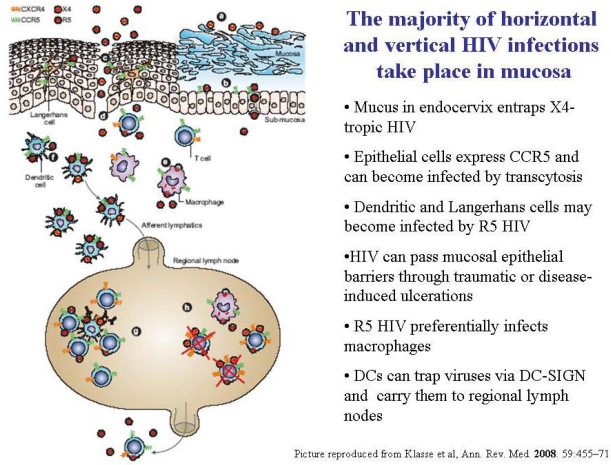
The mucosal scenario of HIV infection. Reproduced from Klasse *et al*., 2008 [69].

The prevalence of R5-tropic HIV can be due to HIV biology as well as to host features; both factors determine the natural history of infection. The mucosal environment is the place where virus-host contact takes place and is where immunity should provide the maximal defense. The majority of HIV infections, both horizontal and vertical transmissions, occur via genital mucosa, via sexual intercourse or child delivery, even if HIV shows a low rate of infection through the genital route [68,69]. Mucosal immunity to infectious agents begins with the physical protection conferred by intact epithelial barriers, then by innate and adaptive immunity (Figure 2).

Pluristratified epithelia protect oral, vaginal, and anal accesses, while monostratified mucosa lines the gut and the endocervix. The presence of mucus, as in the gut and in the cervix, is another physical barrier, which can entrap pathogens and prevent infection by sexually-transmitted or food-borne pathogens. Under the epithelial barrier, genital and intestinal stromal tissues are densely populated with dendritic cells, macrophages, and T cells, which play a role in immune surveillance. The majority of these cell types express CD4 and CCR5 molecules, and therefore offer a large and convenient population of target cells to HIV [66]. In monostratified barriers, such as the columnar cervical epithelium and the gut mucosa, HIV particles may diffuse by transcytosis due to their monostratified structure - these layers offer poor resistance to virus penetration. Conversely, the vaginal mucosa offers a stronger barrier to pathogens, due to the pluristratified structure and to the relatively restricted surface. Microabrasions due to sexual intercourse and concomitant sexually-transmitted infections may weaken or break the vaginal epithelium, facilitating HIV direct diffusion to submucosal tissues [69]. The gut surface, which is considerably larger (up to 400 square meters, *i.e.*, the surface of a tennis court), is monostratified and is therefore less resistant than the vaginal mucosa to microtrauma caused by sexual intercourse. Moreover, submucosal GALT (gut-associated lymphoid tissues) hosts up to 90% of CD4+ and CD8 lymphocytes, making it a more important immune organ than even the blood [69,70]. CCR5 is expressed on intestinal epithelial cells, therefore allowing preferential transmission of R5 viruses via the rectal route. CXCR4 is not expressed on intestinal epithelia, and high levels of SDF-1, CXCR4-blocking ligand, are secreted in the intestinal lumen, thereby hampering the transmission of X4-tropic HIV strains [66].

Mucosal surfaces are characterized by various molecules, including innate mucosal receptors, lipid raft microdomains, and HIV coreceptors [71]. Notably, both CCR5 and CXCR4 receptors are expressed on the genital mucosa. CXCR4 receptors are usually rare, due to down-regulation, and mediated by high local SDF-1 expression [72]. The R5 virus is preferentially transmitted upon its interaction with immune cells residing in the submucosal tissues, such as DC, Langerhans cells, and macrophages; X4 viruses have multiple disadvantages in infecting target cells in deeper mucosal layers. DC may transport HIV to regional lymph nodes where the virus can encounter CD4+ T cells, other targets susceptible to infection by R5 HIV. In the human gut, organized mucosal lymphoid follicles are aggregated to form Peyer’s patches, which are committed to sense microbes and antigens in the lumen and provide a prompt immune response.

The gut epithelium contains specialized sensitive cells, the M cells, which form intraepithelial pockets where submucosal lymphocytes can migrate; M cells capture and deliver samples of intraluminal material by vesicular transport to underlying DCs [73]. Vaginal mucosa lacks organized lymphoid follicles, like those found in Peyer’s pathches and M cells; therefore, DC themselves migrate between epithelial cells, interrupt tight junctions, and obtain samples of foreign material directly from the luminal compartment [74,75]. DC-SIGN, a C-type lectin expressed on submucosal DC in the genital tract, may act as a Trojan horse that facilitates the induction of primary immune responses [59] and at the same time, carries HIV particles to lymph nodes, where naïve T cells will be activated [76]. Further HIV dissemination will proceed from the mucosa-associated lymphatic tissues to other target organs, such as spleen, brain, liver, and lungs, via infected macrophages or T-cells; infected cells can also return to mucosal tissues, through infected mucosal secretions and semen [73,77].

Similarly to infection, the immune response also begins in and spreads from lymph nodes, in the form of plasma cells that secrete neutralizing antibodies, T helper cells that produce cytokines, and cytotoxic lymphocytes. Increased vascular permeability, subsequent to inflammatory stimuli driven by the infection event, facilitates both the local recruitment of macrophages, NK, and T cells, as well as the drainage of IgG molecules *in situ* [71]. Different antibodies isotypes, such as IgG, IgM and IgA, take part in several effector pathways that may protect the host from mucosal infection and clear the virus [78]. Soluble antibodies can compete with HIV for attachment to epithelial cells, participate in opsonization, activate complement-mediated cell lysis, induce antibody-dependent cell cytotoxicity, and mediate transcytosis inhibition [68,77].

## 5. Natural history of individuals carrying anti-CCR5 antibodies

Different types of HIV-blocking antibodies to CCR5 have been isolated from HIV-infected and from HIV-exposed (ESN) subjects.

Antibodies to the HIV binding domain, *i.e.*, the second external loop of the CCR5 molecule, appear in response to HIV infection and block HIV entry through binding competition [79].

Anti-CCR5 antibodies recognizing the first external loop of the protein do not interfere with HIV binding directly, but rather induce coreceptor down-regulation, thus abolishing virus infectivity [80,81]. The generation of anti-CCR5 antibodies to the first external loop, observed in healthy subjects not previously exposed to HIV, could be probably explained by autoimmune phenomena triggered by membrane perturbations unrelated to HIV stimuli, such as the activity of exogenous or endogenous viruses or local inflammation [81,82].

During HIV infection, allo-immune responses to polymorphic surface molecules, such as HLA, may be generated in response to antigens entrapped in HIV particles during budding, or may be elicited via molecular mimicry between gp120, gp41 and host antigens. Allo-immunization can elicit chemokine and CD8 effector cells to HIV in humans [83] and was also shown to be effective to protect monkeys from experimental challenge with infectious SIV [84]. The binding of HIV antigens may induce alterations in host antigens, which are subsequently presented to the immune system in the form of cryptic or uncommon self-epitopes, thereby generating anti-self responses. Responses leading to the generation of anti-self antibodies can also involve idiotype-anti-idiotype networking via an homology/mimicry interplay between HIV and host antigens. This mechanism may lead to the generation of cross-reactive antibodies and to the development of immune complexes, which may entrap viral particles and host antigens and potentially give rise to other uncommon antibody specificities [85].

Antibodies to the first extracellular loop (ECL1 domain) of CCR5 have been only detected in HIV-exposed but uninfected subjects (ESN) and in long-term non-progressing HIV-positive subjects (LTNP), supporting the hypothesis that these antibodies could be involved in HIV protection or in infection control. One clinical study searched for such anti-CCR5 antibodies in 497 subjects, including 85 LTNPs, 70 progressors, 135 HIV+ patients receiving highly active antiretroviral therapy (HAART), and 207 HIV-negative donors [86]. Anti-CCR5 antibodies were isolated in 23% of the LTNP but not in the other subpopulations studied (*P*<.001; Figure 4). Anti-CCR5 Abs were shown to recognize a conformational epitope within the first external loop, and to induce a stable and long-lasting downregulation of CCR5 from the surface of T lymphocytes, thereby inhibiting HIV entry. Receptor internalization was shown to be specifically inhibited by sucrose, but not by filypin or nystatin, nocodazole or cytochalasin D, therefore supporting a specific role for clathrin-coated pits and excluding the caveolae compartments [86]. In addition, CD4+ lymphocytes from the LTNP subset displaying anti-CCR5 Abs were found to be resistant to *in vitro* infection with R5-tropic HIV-1 strains. The level of anti-CCR5 antibodies appeared to be correlated with levels of HIV exposure, being lower in seronegative ESN subjects and higher in seropositive LTNP individuals (0.1% *vs*. 8% of the total antibodies, respectively).

Interestingly, the loss of anti-CCR5 antibodies was observed in the course of clinical follow-up, and this event was significantly associated with clinical progression toward disease in 9 out of 20 LTNP enrolled in the study, some of who experienced a statistically significant increase in viremia and required the resumption of therapy, thus becoming progressors. Strikingly, subjects who retained anti-CCR5 Abs maintained a stable LTNP status without any treatment. According to the finding, the loss of anti-CCR5 Abs was associated with disease progression; toward this observation was strongly supported by the development of AIDS despite antiretroviral therapy in some subjects [86].

The persistence of very low, undetectable levels of HIV replication may provide a continuous antigen boost that does not result in a strong generalized immune activation, similar to what is observed in the course of natural latent viral infections (e.g., herpes viruses) or in food-delivered antigens and/or vaccines, which may establish tolerance but also retain their antigenic potential [70,87]. In the lucky subset of ESN and LTNP individuals able to control HIV, physiological and immunological conditions might have established a positive feedback loop that maintains undetectable levels of virus replication and a suitable antigen presentation on one hand; and on the other hand, long-lasting responses that are able to block HIV through its major coreceptor provide a key mechanism for fighting HIV replication [85]. Another key point in the study is the observation that the viral phenotype in LTNPs carrying anti-CCR5 antibodies did not shift in the presence of such antibodies, thus confirming that the selective pressure of CCR5 inhibitors does not induce a change of viral phenotype *per se*, as already reported in a monkey model [88]. In addition, anti-CCR5 antibodies were not found to induce any apparent alterations in immune function, as demonstrated by the continued health of subjects who retained anti-CCR5 antibodies; both these findings provide arguments against theoretical concerns about CCR5 targeting with specific antibodies.

## 6. Strategies for CCR5 targeting

**Figure 3.**
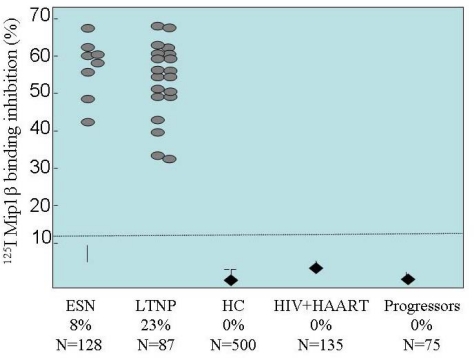
Anti-CCR5 antibodies to the first extracellular loop, isolated in various cohorts of HIV-exposed or HIV-infected, Long-Term non progressing people. Modified from Pastori *et al*., 2006 [86].

**Figure 4.**
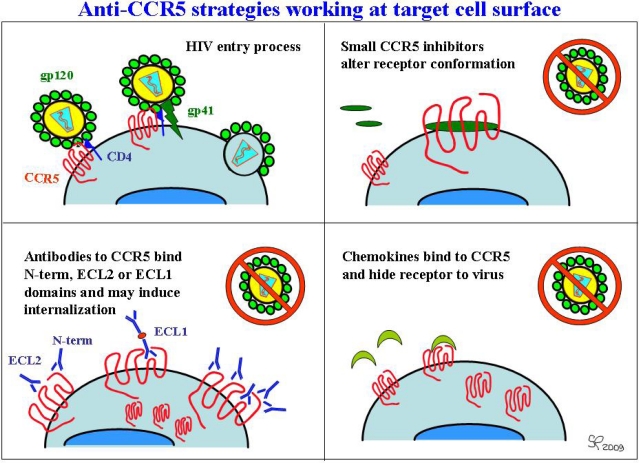
Current approaches to extracellular CCR5 blocking.

Due to its features and its natural history, CCR5 is a key target in HIV therapy and prevention. CCR5-fostered therapeutic approaches to block HIV infection to date, including small molecule inhibitors, chemically modified ligands, and anti-CCR5 antibodies, have shown their antiviral properties in cell-based tests and in *in vivo* trials [56,89,90]. These approaches, shown in Figure 3, can be defined as “extracellular”; other approaches, still the subject of preclinical research, target CCR5 expression from an “intracellular” point of view; for example, taking advantage of drugs such as rapamycin [91] or statins [92], which prevent CCR5 surface expression. Interfering mRNAs (siRNAs) [93] and ribozymes [94] have also been shown to interfere with CCR5 expression. Other methods being developed, such as “intrabodies” (single-chain, intracellular antibodies) [95] or “intrakines” (intracellular chemokines) [96], also aim at trapping CCR5 within cells, thus preventing its surface expression and/or its recycling [2].

### 6.1. Small molecule inhibitors

The binding of HIV to CCR5 occurs after the CD4-gp120 interaction, and usually triggers a conformational change in the HIV envelope, which allows gp41 activity and promotes virus-cell fusion. Small molecule antagonists of CCR5 that bind to CCR5 within a pouch created by the seven membrane-spanning hydrophobic helices, stabilize the receptor conformation and prevent HIV binding to the extracellular domains, namely to the N-terminus and ECL-2, thereby blocking gp41-mediated fusion and subsequent viral replication [89]. CCR5 antagonists do not act on CXCR4, so their use is not recommended in patients hosting X4 or R5/X4 dual tropic viruses. Usually X4 and dual-tropic virus isolates appear later in the course of infection, and their appearance is usually associated with a faster decrease in CD4+ T cells count and the progression towards symptomatic disease.

Two joint clinical trials (MOTIVATE one and two) have demonstrated the efficacy and safety of maraviroc, which is currently approved for use in treatment-experienced patients; other drugs are currently under preclinical or clinical development. After 48 weeks, patients receiving maraviroc showed at least a 1.5 log reduction in viral RNA copies/mL - significantly higher than the control - and more than 40% of patients in all groups of treatment showed virus replication levels of <50 copies/mL. Moreover, treated patients also displayed a significantly greater increase in CD4+ cell count than the controls. Conversely, a consistent percentage of treatment failure was observed, mostly due to the emergence of dual-tropic or X4 viral strains that were not blocked by antiviral therapies associated with the study drug [56].

HIV is notorious for its ability to overcome immune defenses ─ and antiretroviral therapy ─ through Darwinian selection, *i.e.*, by random gene mutations and selection of drug-resistant viral strains. At the molecular level, resistance to small inhibitors involves point mutations in some *env* domains, such as the variable V3 loop and C2-V5 mutations; V3 mutations by themselves were found necessary but not sufficient for resistance [97,98]. Changes in the V3 region can strengthen the *env* interaction with the N-terminal domain of CCR5, while the conformation of the first and second extracellular loops, regions, altered by most CCR5 inhibitors, do not affect *env* binding and viral entry [99]. Indeed, CCR5 blockage could offer the virus the obvious alternative to use CXCR4 molecules to bypass the drug effect. A switch towards a X4 phenotype has been shown to occur *in vitro* and *in vivo*, however, X4 viruses appear to be selected in control as well as in cell cultures treated with inhibitor molecules, suggesting that the viral genetic drift may occur independently of drug-induced selection, e.g., as an adaptation to infect PBMC cells [89]. Probably, MOTIVATE patients already hosted X4 virus strains at the time of enrollment; according to other *in vitro* assays, the R5 viruses tended to retain their phenotype under inhibitor selection, probably because the original R5 viruses were less sensitive to a selective environment than the newly arisen ones [89].

Two major mechanisms have been described to explain HIV resistance, the competitive and the non-competitive (allosteric) mechanisms. Competitive resistance appears as a shift in the drug IC50; higher drug doses still achieve a 100% inhibition. Allosteric inhibition, conversely, does not change IC50 values, but reduces the maximal viral inhibition below 100%, which is insensitive to further drug addition. In competitive resistance, viruses most efficiently use CCR5 molecules that are still free from drugs, while in allosteric resistance, new variant viruses become able to use drug-bound coreceptor as well as free-CCR5 molecules [89].

Various studies and clinical observations support the idea that the R5 to X4 switch is a complex event, which is not directly associated with the use of CCR5 inhibitors or other anti-retroviral drugs. The coreceptor switch was observed in approximately 50% of HIV-infected patients carrying sub-type B viruses, but progression to AIDS also occurred in patients hosting uniquely the R5 virus with various functional changes in multiple *env* domains [100,101]. More strikingly, HIV entry efficiency via CCR5 was found to improve in patients that remained R5-positive only, whereas it declined in viruses isolated from patients carrying R5X4 viruses [102]. This apparently paradoxical finding can be explained by results of other studies, which depicted dual-tropic R5X4 viruses as an heterogeneous population, in which “dual-R” (CCR5-preferering) or “dual-X” (CXCR4-preferring) viruses can be distinguished [103].

Small CCR5 inhibitors do not always induce cross-resistance to other antiviral drugs, either acting via CCR5, such as antibodies or modified chemokines, and to drugs endowed with other mechanisms of action, such as protease inhibitors or RT-inhibitors [90]. In some cases, the acquisition of resistance does not appear to compromise viral “fitness”, *i.e.*, its replicative ability; in other cases, once the selective agent is no longer administered, the virus phenotype reverts to sensitivity, showing that that *in-vitro* resistance can carry a fitness cost [97]. However, *env* glycoproteins are under continual selection pressure of neutralizing antibodies (NAbs) *in vivo* [104]; when small inhibitors are administered, HIV undergoes a double selective pressure, which may impose functional constraints on virus variability. For example, a virus variant able to evade an inhibitor can maintain sensitivity to a Nab. According to a study evaluating mutant HIV strains resistant to small inhibitors, viruses resistant to vicriviroc or AD101 also showed cross-resistance to other small inhibitors, such as aplaviroc or maraviroc. However, they retained sensitivity to other antiretroviral drugs, such as reverse transcriptase inhibitors, protease inhibitors and fusion inhibitors, such as enfuvirtide, which are endowed with mechanisms of action independent of CCR5 [105]. Strikingly, such escape mutants were sensitive to the chemokine ligand PSC-RANTES, to neutralizing mAbs, such as the humanized antibody PRO140, and to sera from HIV-infected people [105]. These findings show that *env* mutations conferring resistance to CCR5 inhibitors do not necessarily affect the efficacy and safety of other antiviral strategies, particularly the activity of humoral immunity ─ chemokines and antibodies ─ either natural, elicited or administered.

### 6.2. Monoclonal antibodies to CCR5

Humanized monoclonal antibodies recognizing the CCR5 extracellular N-terminal and/or second extracellular loop domains have been developed and were able to compete with gp120 binding [2]. When assayed *in vitro* at nanomolar concentrations, PRO140 was able to block HIV strains belonging to different clades, both in primary macrophages and in PBMC [106]. PRO140 and another mAb, HGS004, have been tested in HIV-infected subjects [107,108]. PRO140 was able to inhibit HIV without blocking CCR5 response to chemokines, whereas HGS004 prevented both viral infection and chemokine signaling. Notably, antibodies and small molecule antagonists do not share the same mechanism and site of action; therefore, their activity may be synergic or contrasting, and no cross-resistance has been observed [2].

### 6.3. Engineered chemokines

Natural chemokines have been found to prevent HIV binding to its coreceptors, due to steric hindrance or competition for binding sites or to receptor internalization. Conditions which induce sustained production of beta chemokines (CCL3/MIP-1alpha, CCL4/MIP-1beta, CCL5/RANTES) have been associated with a lower risk of HIV transmission; similarly, natural mutations that enhance production of SDF-1, and therefore increase competition for the CXCR4 coreceptor, were shown to play a protective role in HIV infection [72,83]. Due to the short half-lives (<10 min) of natural chemokines, various N-terminally modified chemokines have been synthesized and tested for the ability to prolong or shorten CCR5 internalization [2]. AOP-RANTES (aminooxypentane-RANTES) inhibits different HIV strains infecting PBMC through the induction of gamma-IFN and the parallel reduction of IL-10 expression [109]. Its mechanism of action relies on coreceptor internalization and a long-lasting inhibition of CCR5 recirculation [110]. PSC-RANTES ([N-nonanoyl, des-Ser1[L-thioproline2, L-cyclohexylglycine3]) is a strong CCR5 agonist and a highly potent inhibitor of HIV entry *in vitro*; it confers full protection from R5-mediated infection in an animal model of vaginal transmission, due to long-lasting receptor internalization. However, CCR5 binding by CCL5/RANTES derivatives is also associated with mucosal inflammation, a phenomenon which would enhance HIV infection [111]. Pharmaceutical research and development is presently striving to identify new derivatives that can be produced in inexpensive expression systems by the fermentative industry without requiring post-synthetic chemical modifications. Moreover, ideal CCL5/RANTES derivatives should also separate HIV-inhibition activity from CCR5 signaling, in order to prevent *in vivo* potentially harmful pro-inflammatory activity [111,112]. It should also be remembered that engineered chemokines, similarly to small molecule inhibitors, were shown to exert selective pressure on CCR5-tropic viruses, with consequent development of R5 inhibitor-resistant HIV strains and finally, leading the way to dual-tropic or X4-tropic virus shift [113].

### 6.4. Anti-CCR5 vaccination

Anti-CCR5 antibodies recognizing the first external loop of the protein do not interfere with HIV binding directly, but induce coreceptor down-regulation, thus eliminating virus infectivity [81,114]. Such rare antibodies raise questions about their genesis, which may be natural (via genetic mechanisms) rather than elicited by some still undetectable HIV-unrelated or very low level antigenic stimulation. However, despite some still open questions, some vaccination experiments have successfully elicited anti-CCR5 auto-antibodies and have investigated both *in vitro* and *in vivo* the immune protection they confer.

Immunization experiments and *in vitro* studies of elicited antibodies were performed by Chain *et al*. [115], who immunized rabbits with chimeric peptides encoding a very short fragment of the N-terminal sequence of CCR5 (Met1-Ser7 or Asp2-Ser7), and a T-specific peptide from *Tetanus* toxoid. T-specific CCR5 epitopes were not included in the immunogen, in order to prevent the development of host autoimmune responses. The immunization generated a strong antibody response. Binding experiments to N-terminal and full-length CCR5 suggest that only a small percentage of the antibodies elicited by immunization were able to bind CCR5; nevertheless, anti-CCR5-specific antibodies blocked HIV infection in macrophages *in vitro*. In a subsequent study, Devito *et al*. [116] èerformed a long-term immunization with intranasal DNA prime followed by a peptide booster immunization. Delivered antigens were from gp120 V3 loop, gp41 (MPER peptides containing the ELDKWAS epitope) and CCR5-ECL domain (aa.168–185). The vaccination schedule elicited specific IgG and IgA in sera and in mucosal secretions (intestinal, vaginal and lung) in immunized mice. More interestingly, long-term IgG and IgA responses were still observed 12 months after boosting - both in serum and in mucosal secretions. HIV-1-neutralizing antibodies were still detected in serum 12 months after boosting. According to this study, intranasal DNA prime followed by one peptide/L3 adjuvant booster immunization, ─ but not vice versa ─ induced long-lasting neutralizing antibodies and B memory cells to poorly immunogenic, conformational epitopes. Barassi *et al*. [81] generated chimeric immunogens containing a CCR5 peptide from the first excellular domain (Tyr89-Trp102) in the context of the capsid protein of flock house virus, a conformation-constrained expression system [117]. When administered to mice by systemic or mucosal route, the immunogens elicited anti-CCR5 IgG and IgA both in sera and in vaginal fluids. Similarly to HIV-exposed seronegative individuals, mice producing anti-CCR5 autoantibodies expressed significantly reduced levels of CCR5 on the surfaces of CD4+ cells from peripheral blood and vaginal washes. *In vitro* studies showed that murine IgG and IgA (i) specifically bound human and mouse CD4+ lymphocytes and the CCR5-transfected U87 cell line; (ii) down-regulated CCR5 expression of CD4+ cells from both humans and untreated mice, (iii) inhibited CCL4/MIP-1beta chemotaxis of CD4+ CCR5+ lymphocytes, and (iv) blocked HIV R5 strains. Finally, Pastori *et al*. [118] performed a peptide-scanning assay on a panel of synthetic peptides spanning the CCR5-ECL1 region; the resulting peptides were assayed with a pool of natural anti-CCR5 antibodies and used to immunize mice and chickens. Further structural characterization of the peptides was provided by NMR spectroscopy and molecular dynamics simulations. Amino acid substitutions in positions 95 and 96 (Ala95-Ala96) increased antibody-peptide binding compared to the wild-type peptide (Asp95-Phe96). The Ala95-96 peptide was able to induce antibodies, both in mice and chickens, which displayed biological activity at very low concentrations. Strikingly, chicken antibodies to the Ala95-96 peptide specifically recognized human CCR5 molecules, down-regulated receptors from lymphocytes, inhibited CCR5-dependent chemotaxis, and prevented infection by several R5 viruses, displaying IC50 values lower than 3 ng/ml. NMR spectroscopy and molecular dynamics simulations confirmed the high level of flexibility of the isolated epitopes and suggested that A95-A96 substitutions led to a slightly higher tendency to generate helical conformations combined with a lower steric hindrance of the side chains in the peptides. The different structural behavior of the mutagenized loop may account for a better molecular structural organization, allowing the induction of the fittest antibodies. Optimized antibodies recognized and bound native CCR5 with higher affinity and displayed enhanced biological activity.

Other *in vivo* studies coupled immunization studies with *in vivo* challenges to vaccinated animals to evaluate whether a break in B-tolerance had been achieved and the extent of immune protection conferred by the tested immunogens. Chackerian *et al*. [119] used the N-terminal domain of pigtailed macaque CCR5 fused to streptavidin, which when conjugated at high densities to bovine papillomavirus major capsid protein L1 virus-like particles induces high-titer anti-CCR5 IgG that blocks infection by CCR5-tropic simian-human immunodeficiency virus (SHIV) *in vitro*. No decline in the number of CCR5-expressing T cells was detected in immunized animals. In SHIV-challenged macaques, viral loads and time to control of viremia were significantly decreased (relative to controls), indicating the possibility that CCR5 autoantibodies contributed to the control of viral replication. Bogers *et al*. [120] assayed a vaccine consisting of three extracellular peptides of CCR5, an N-terminal HIV gp120 fragment generated in transgenic plants, and recombinant simian immunodeficiency virus p27. They were linked to the microbial heat shock protein HSP70 used as a carrier, and the vaccine was administered by mucosal and systemic routes. Vaginal challenge with SHIV infected all macaques, but showed a significant variation in viral loads between the animals, and the virus was cleared in five of nine immunized animals. Misumi *et al*. [121] adopted synthetic cyclic peptides from the second external loop (Arg168 to Thr177) to induce anti-CCR5 antibodies in cynomolgus macaques. The immunization with cDDR5-conjugated multiple-Ag peptide (cDDR5-MAP) induced long lasting anti-cDDR5 antibodies reacting with both human and macaque CCR5 molecules, which were able to suppress infection by the R5 HIV-1 laboratory isolate (HIV JRFL), R5 HIV-1 primary isolates (clade A:HIV 93RW004 and clade C:HIV MJ4), and a pathogenic simian/HIV (SHIV SF162P3) bulk isolate *in vitro*. After SHIV challenge, cynomolgus macaques showed an attenuated acute infection and a lower viral load than unvaccinated control animals.

According to *in vitro* and *in vivo* findings, immunization does elicit antibodies endowed with neutralizing properties, showing that B-tolerance can be effectively broken; despite none of the immunogens assayed *in vivo* being able to confer full protection from the virus challenge; infection of vaccinated subjects was milder than in the controls and virus control was achieved in most subjects. Finally, *in vitro* studies also demonstrate that conformational changes in the CCR5 protein, together with host factors, have the potential to modulate protein immunogenicity *in vivo* and could also play a role in the natural resistance to HIV infection.

## 7. Conclusion and perspectives for a vaccination intervention

CCR5 is a key player in HIV entry and many attempts to prevent its role in infection have been developed and assayed. The clinical use of small CCR5 inhibitors has proven the feasibility and the efficacy of CCR5 targeting, but it has also raised concerns about the safety of this approach: R5-resistant HIV strains have been isolated in cell cultures and in patients receiving maraviroc and other CCR5 inhibitors [56,89,105]. The use of humanized monoclonal antibodies has been shown to be effective, safe, and long-lasting in HIV-infected patients, suggesting that passive immunization may also offer therapeutic advantages [107,108]. The use of engineered chemokines induces receptor down-regulation, therefore preventing the binding of CCR5 to HIV. However, despite its effectiveness, this approach might be associated with adverse inflammatory events *in vivo* [111]. An HIV vaccine remains the most expected goal to be accomplished in HIV research, proving its value both in therapeutic intervention and in prevention [122]. Vaccination may offer long-lasting protection with few administrations, an alternative in many geographical and social contexts where other forms of prevention for sexually-transmitted diseases could be impractical or rejected [69].

Anti-CCR5 vaccination is an innovative anti-HIV strategy, which could provide effective protection or safe containment of the spread of the virus. Indeed, the feasibility of anti-CCR5 vaccination has been already demonstrated by two groups of naturally occurring CDC5-deficient people. Individuals deprived of CCR5 receptor by genetic deletion [30,123,124] and those carrying naturally occurring anti-CCR5 antibodies that down-regulate the receptor *in vivo* [82,86,114], were found to be healthy and very resistant to HIV-infection. Very importantly, such natural anti-CCR5 antibodies were observed in sera and in mucosal fluids from individuals who remained uninfected despite repeated, unprotected, sexual exposure to HIV, and in infected individuals with long-term, asymptomatic infection. The finding that both ESN and LTNP subpopulations exert a high and durable control on the virus supports the hypothesis that natural anti-CCR5 antibodies could be associated with protection. This concept is further strengthened by the good health and immune status shown by the LTNP cohort, suggesting that long-lasting CCR5 down-regulation is not harmful; conversely, the loss of anti-CCR5 responses experienced by some patients in cohort follow-up was associated with a decline in virus control [86]. These findings are noteworthy, because genetic CCR5 deletion is associated with an increased susceptibility to some viral and bacterial pathogens [41]. Moreover, anti-self immunity was one of the mechanisms evoked to explain the generation of natural anti-CCR5 antibodies [85] and a possible adverse event associated with anti-CCR5 vaccination [2]. Conversely, CCR5 targeting could offer therapeutic advantages just in some autoimmune-based diseases, such as rheumatoid arthritis [47], or in transplantation therapy - all situations where chemokine signaling and cell recruitment are immune mechanisms sustaining tissue damage [50]. Another key finding from the follow-up of the LTNP cohort was the lack of a R5-to-X4 shift, a fact supporting the safety of antibody-mediated coreceptor targeting [86]. This is a key point to be considered due to the concerns raised by the therapeutic use of small CCR5 inhibitors, which are prone to developi of *in vitro* and *in vivo* drug resistance and might favor the selection of dual-tropic or X4-tropic virus strains [88,89,125]. Indeed, immunization experiments performed in animals have shown that anti-CCR5 antibodies can be obtained *in vivo*, provided that suitable vector systems are used, either to break B-tolerance to the self CCR5 antigen and to constrain the ECL1 peptide (*i.e.*, the target domain of natural anti-CCR5 antibodies) in a conformation similar to the naturally occurring, immunogenic one [81,118]. Moreover, anti-CCR5 antibodies elicited by mucosal route were shown to be long-lasting and promptly re-boosted upon immunization, in sera and most importantly in mucosal fluids, demonstrating the feasibility of local immunity at major portals of HIV entry [81].

Taken together, all of the findings we have reviewed here support the significance of interventions aimed at targeting the CCR5 molecule as a principal HIV coreceptor. Among all the strategies now available or under development, naturally occurring anti-CCR5 antibodies show the therapeutic potential to provide durable, effective, and safe systemic, and especially, local immunity to HIV. From follow-up studies and immunization experiments, antibody-mediated CCR5 targeting has been shown to be not only feasible but also well tolerated. Together with other immune-modulating strategies, this unconventional approach could open unprecedented scenarios not only in HIV vaccinology, but possibly also in the treatment and prevention of other disorders where harmful pro-inflammatory responses can develop.
